# Chronic diseases alter the platelet rheostat to promote hyperreactivity and thrombosis

**DOI:** 10.1172/JCI194082

**Published:** 2025-08-15

**Authors:** Roy L. Silverstein

**Affiliations:** Department of Medicine, Medical College of Wisconsin and Versiti Blood Research Institute, Versiti Blood Center of Wisconsin, Milwaukee, Wisconsin, USA.

## Abstract

Platelet hyperreactivity, defined as enhanced sensitivity to activation in response to classical agonists, contributes to the increased risk of arterial thrombosis associated with chronic inflammatory diseases. In this issue of the *JCI*, Kong and colleagues used an unbiased proteomic approach to identify elevated SEC61B in platelets from patients with diabetes and from hyperglycemic mice. Typically, SEC61B participates in protein transport within the endoplasmic reticulum (ER), but it can also act as an ion channel that allows calcium to leak from ER to cytoplasm. The authors showed that elevated SEC61B expression caused increased calcium leak, elevated basal cytoplasmic calcium concentrations, and platelet hyperreactivity. In vitro and in vivo pharmacological interventions to alter calcium homeostasis through this pathway affected platelet reactivity. The results of this work are consistent with those of previous studies showing that platelets from patients with chronic diseases behaved differently than those from healthy participants. These findings identify potential disease-specific targets to prevent and treat arterial thrombosis.

## Platelet hyperreactivity in chronic disease

Chronic inflammatory diseases, including diabetes, obesity, cancer, atherosclerosis, and autoimmune disorders, are known to increase the risk of an arterial thrombotic event such as a heart attack and stroke. Platelet hyperreactivity, defined as enhanced sensitivity to activation in response to classical agonists, such as thrombin, collagen, thromboxane, and ADP, is thought to play an important role in this risk. Although most of our knowledge of platelet physiology and pharmacology is based on studying normal platelets from healthy humans, an emerging concept is that platelets may respond differently in the setting of chronic disease. For example, Cameron et al. (1) showed that the activation of the atypical redox-sensitive MAP kinase ERK5 in platelets, which was not previously associated with platelet activation, triggered infarct expansion in a mouse model of acute myocardial infarction and ischemia/reperfusion injury.

Several mechanisms have now been shown to promote platelet hyperreactivity in the context of chronic disease. The altered environment in these settings can generate agonists for platelet receptors beyond those typically considered part of classical hemostasis. These receptors recognize products of both the innate and adaptive immune system, and danger-associated molecular patterns (DAMPs) produced during inflammation, hyperglycemia, hyperlipidemia, and tissue injury. Among these DAMPs are oxidized low-density lipoprotein (oxLDL), apoptotic cells, cell-derived microvesicles, advanced-glycated proteins, and cancer cell–derived mucins. Our research has shown that the platelet scavenger receptor CD36, which recognizes oxLDL (2), AGE proteins (3), and microvesicles (4), increased the sensitivity of platelets to classical agonists and promoted arterial thrombosis in mouse models of diabetes, hyperlipidemia, and chronic systemic inflammation. Mechanistically, CD36 signals when engaged with its ligands by assembling a multiprotein complex of membrane and intracellular proteins (5) that activate specific src-family kinases and MAP kinases (6). These signals result in production of intracellular ROS that drive platelet activation (7).

Products of the innate immune system released during inflammation, including S100A proinflammatory neutrophil peptides (8), ATP (9), and oxylipins (10), can also activate specific receptors on platelets, such as CD36, P2X1, and G protein–coupled receptors, respectively. Additionally, platelets express the Fcγ receptor (FcγR) γ chain, a small surface protein containing an intracellular immunoreceptor tyrosine-based activation motif (ITAM) that associates with other receptors to transduce activating signals through src-family kinases (11). Most prominent among these is FcγRII, which binds circulating pathogenic immune complexes such as those seen in the syndrome of heparin-associated thrombocytopenia with thrombosis. The C-type lectin receptor CLEC2 is also expressed on platelets (12). It contains a partial ITAM domain and also interacts with FcγR γ chain to activate platelets in response to podoplanin, a mucin found on tumor cells and lymphatic vessels that may contribute to the thrombotic risk associated with cancer and tissue injury.

Some of these disease-associated receptors may act independently of classical pathways, whereas others appear to work in an additive or synergistic manner with classic agonists. We have shown that oxLDL signaling, through CD36, sensitized platelets to aggregation induced by low or subthreshold doses of ADP and synergized with collagen to enhance surface expression of phosphatidylserine, which promotes thrombin generation on the platelet surface (7). This augmentation of platelet activation may in turn diminish a patient’s response to currently available antiplatelet therapies.

In the current issue of the *JCI*, Kong and coauthors make an important contribution to the field by linking disordered platelet calcium homeostasis to diabetes-related platelet hyperreactivity (13). They studied isolated platelets from 79 human participants with known or suspected coronary disease and compared platelets from individuals with type 2 diabetes mellitus with those from individuals without diabetes mellitus. Using a sensitive, unbiased proteomic platform they identified proteins expressed at higher level in the diabetic platelets. Gene ontogeny analysis showed that most of these changes were in pathways related to oxidative stress, cytoskeletal dynamics, or ER stress. However, they focused on one protein, SEC61B, because it was the sole protein associated with circulating levels of fructosamine, a nonenzymatically glycated protein that reflects glucose levels over the prior 2–3 weeks. Fructosamine is a useful biomarker of poor glycemic control and a risk factor for atherothrombosis. Importantly, the authors validated their initial findings by observing increased SEC61B in platelets from a second cohort of patients with diabetes as well as in platelets and megakaryocytes from mice with hyperglycemia.

## Calcium signaling in platelets

SEC61B is a component of the SEC61 translocon, a heterotrimeric protein complex that traffics polypeptides through the endoplasmic reticulum (ER). Although platelets have little capacity for protein synthesis, they have an extensive ER known as the dense tubular system that serves a critical function in platelet activation by storing calcium at millimolar concentrations and releasing it into the cytoplasm in response to classical platelet agonists (14). This cytoplasmic calcium spike activates a set of calcium-dependent reactions that trigger platelet shape change, spreading, aggregation, secretion, and thromboxane generation. The millimolar calcium concentration in the ER and nanomolar concentrations in the cytoplasm are maintained by SERCA proteins that actively pump calcium from cytoplasm to ER (15).

Upon platelet engagement with classical agonists, phospholipase C is activated, which leads to production of inositol triphosphate (IP3) and diacylglycerol (DAG). IP3 engages with the IP3 receptor on the ER membrane to rapidly release calcium into the cytoplasm. Calcium also enters the cytoplasm from the extracellular space through a process called store-operated calcium entry, triggered by the ER protein STIM1 that senses calcium depletion in the ER and activates the surface membrane calcium channel ORAI1 (14). Additionally, TRPC6 and P2X1 are calcium channels on the platelet surface activated by DAG and ATP, respectively, and contribute to calcium influx during platelet activation. Within this complex homeostatic system, SEC61 functions as a calcium channel in the ER, allowing calcium to leak back into the cytoplasm (14).

## Hyperglycemia alters platelet homeostasis

It is reasonable to hypothesize that the perturbation of any element of this system could affect platelet activation. In this scenario, changes that increase basal cytoplasmic calcium levels could lead to platelet hyperreactivity. Indeed, platelets from patients with diabetes have been shown to have increased calcium levels and increased activation of PKC (a calcium-dependent enzyme) along with activation of IRE1, which is an indicator of ER stress (16, 17). Thus, the authors in Kong et al. (13) hypothesized that increased SEC61B levels would increase calcium leak, raise cytoplasmic calcium levels, and position the platelets to be more responsive to activating signals. Using multiple pharmacologic and genetic approaches to modulate the SEC61 channel, SERCA proteins, and ER stress, they were able to link increased SEC61B expression to ER stress, increased calcium leak, and increased cytosolic calcium in platelets in vitro and in vivo. They also showed that increasing cytosolic calcium through increased expression of SEC61B or inhibition of SERCA led to increased platelet activation in response to low doses of thrombin. Conversely, pharmacologic inhibition of the SEC61 complex decreased platelet cytoplasmic calcium concentrations and inhibited platelet aggregation in vitro and blunted thrombus formation in vivo in hyperglycemic mice. Mechanistically, it remains to be determined how hyperglycemia and ER stress lead to increased SEC61B expression in platelets. This work demonstrates how the complex calcium homeostatic system in platelets can be influenced by chronic diseases and potentially targeted to limit platelet hyperactivity under pathogenic conditions.

Another important finding from proteomic studies by Kong et al. was the discovery that, in response to low-dose thrombin, the releasate from platelets isolated from patients with diabetes included over 40 proteins that were not seen in the releasate from individuals without diabetes (13). Some of these proteins may contribute to hyperactivity and thrombosis. The mechanisms for this differential protein release are unknown but could relate to differential sorting of proteins into platelet granules in response to oxidative stress or other posttranslational modifications. Differences may also arise in alternative lysosome-derived compartments due to enhanced autophagy or mitophagy associated with chronic disease.

The elegant studies of Kong et al. (13) raise many interesting unanswered questions, including whether the secretome differences observed in platelets from individuals with diabetes can be quantified and validated in other cohorts and which, if any, of the proteins and pathways identified have functional consequences. Additional in vivo thrombosis models will also help clarify the translational importance of SEC61B upregulation. Dissecting the molecular mechanisms linking ER stress to upregulated SEC61B may also identify additional therapeutic targets for preventing thrombosis in diabetes and other chronic conditions associated with ER stress.

## Conclusions

Platelet activation follows precise coordination of multiple complex, intersecting signaling pathways (18). It is therefore essential to approach the effects of chronic disease on platelet reactivity as a systems biology problem. The work of Kong et al. (13) adds to a growing body of evidence suggesting that there may be common mechanisms, including ER stress (19), altered calcium homeostasis, redox stress (7), and autophagy (20), by which pathological conditions promote platelet hyperactivity. Our group has found that ROS generated downstream of CD36 oxidized allosteric cysteine residues in src-kinases to enhance their enzymatic activity and modified active site cysteines in phosphatases to inhibit their activity (7). These results suggest that the delicate balance of kinase and phosphatase activities can be tipped to promote platelet activation and thrombosis. Redox stress and elevated ROS levels in platelets may also blunt SERCA activity, thereby altering calcium homeostasis. Recent studies by Young and colleagues have shown that oxidative stress induced cysteine cross-links in proteins containing free cysteines and that this impaired protein function by reducing protein mobility and limiting intracellular protein trafficking (21).

This growing body of work supports the concept that healthy resting platelets have systems in place that act as a functional rheostat to optimize hemostatic function by providing the right balance of enzymatic activity, calcium levels, redox state, energy homeostasis, and proteostasis. Disruption of this so-called “Goldilocks” state under chronic pathogenic conditions can alter these settings to promote thrombosis or bleeding. Work by Kong et al. (13) and others has identified potential targets to reset this rheostat and prevent and treat arterial thrombosis in the context of hyperglycemia or other diseases.

## Version 1 08/15/2025

Electronic publication
